# Indiana

**Published:** 1896-12

**Authors:** 


*Indiana.* While tuberculosis seems quite prevalent through this State the appropriation to the Live-stock Commission has been cut down to $4000, a meagre allowance for such important work, and an exhibition of false economy that ranks well with the action of the legislators in the Empire State at their last session. There will be a rude shaking up in this matter in the near future in these States, and the losses to those engaged in animal industry will be a sad reflection on the intelligence of their lawmakers.

